# Decreasing amyloid toxicity through an increased rate of aggregation†
†Electronic supplementary information (ESI) available. See DOI: 10.1039/c6cp06765d. The data supporting the publication can be accessed at https://doi.org/10.17863/CAM.6653.



**DOI:** 10.1039/c6cp06765d

**Published:** 2016-11-30

**Authors:** Silvia Sonzini, Helen F. Stanyon, Oren A. Scherman

**Affiliations:** a Melville Laboratory for Polymer Synthesis, Department of Chemistry, University of Cambridge , Lensfield Road , Cambridge CB2 1EW , UK . Email: oas23@cam.ac.uk

## Abstract

Amyloid β is one of the peptides involved in the onset of Alzheimer's disease, yet the structure of the toxic species and its underlying mechanism remain elusive on account of the dynamic nature of the Aβ oligomerisation process. While it has been reported that incubation of Amyloid β (1–42) sequences (Aβ42) lead to formation of aggregates that vary in morphology and toxicity, we demonstrate that addition of a discrete macrocyclic host molecule, cucurbit[8]uril (CB[8]), substantially reduces toxicity in the neuronal cell line SH-SY5Y. The macrocycle preferentially targets Phe residues in Aβ42 complexing them in a 2 : 1 fashion in neighboring peptide strands. A small but significant structural ‘switch’ occurs, which induces an increased aggregation rate, suggesting a different cell-uptake mechanism for Aβ42 in the presence of CB[8]. Dramatically increasing the rate of Aβ42 aggregation with CB[8] bypasses the toxic, oligomeric state offering an alternative approach to counter Alzheimer's disease.

## Abstract 2

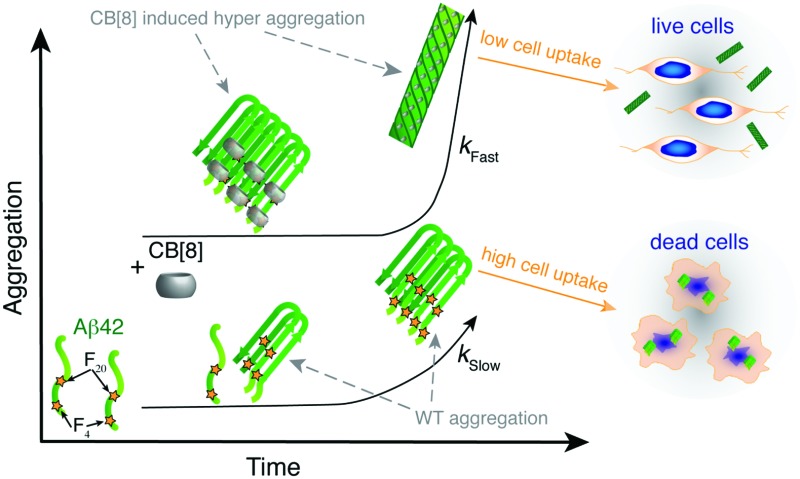
While it has been reported that wild type Amyloid β (1–42) aggregates are highly toxic, we demonstrate that addition of a discrete macrocyclic host molecule, cucurbit[8]uril, increases the aggregation rate of the peptide but substantially reduces its toxicity.

## Introduction

**Fig. 1:**
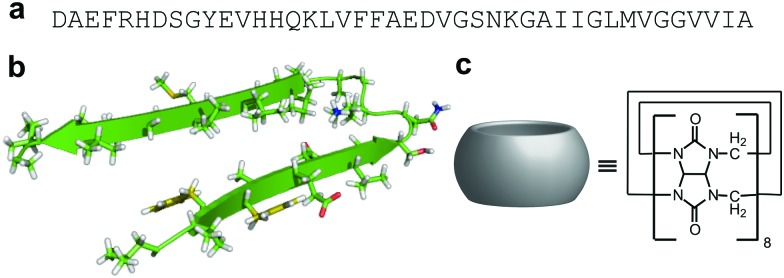
Aβ42 and CB[8] structure and analyses of their binding interactions. (a) Aβ42 sequence, one letter code. (b) Model representation of Aβ42 hairpin produced from PDB 2BEG6. (c) CB[8] cartoon and molecular formula.

The onset of Alzheimer's disease involves the over-expression of two pathological amyloid proteins, namely, Amyloid β (Aβ), found *in vivo* both as soluble oligomers and as fibrillar deposits in senile plaques, and hyperphosphorylated *tau* aggregates, observed in neurofibrillar tangles.^1,2^ Aβ varies in length from 39 to 43 amino acid residues (Fig. 1a) and arises from β- and γ-secretase enzymatic cleavage of the amyloid precursor protein.^3^ Amyloid β_1–40_ (Aβ40) and Amyloid β_1–42_ (Aβ42) are considered major hallmarks of Alzheimer's disease, the latter being more prone to aggregation and generally more toxic.^1,4,5^ Disease progression is related to misfolding and aggregation of the peptide fragment into oligomers with β-arch structural motifs. (Fig. 1b)^6^

It has been reported that Aβ oligomers of different size and morphology, such as dimers,^7,8^ tetramers,^9,10^ pentamers,^4^ dodecamers^11^ and Aβ-derived diffusible ligands^12^ are connected to neuronal impairment. Nevertheless, as the soluble oligomeric aggregates are in constant equilibrium with each other, determining both their structure and their mechanism of interaction with neuronal cells has remained elusive. It has been suggested that elevated toxicity of small, soluble oligomers compared to mature fibres may be related to their higher diffusion through tissues and into various cellular compartments,^2,13^ enabling both receptor-mediated and non-receptor-mediated membrane interactions. Furthermore, the lower toxicity of amyloid fibrils suggests that many of the toxic structural elements are inaccessible in the fibre core.^2,13^ The higher toxicity of oligomers over fibres was recently corroborated by Cohen *et al.*, whereby a chaperone was shown to inhibit the formation of small aggregates and redirect the aggregation pathway towards fibre formation, thus decreasing Aβ42 toxicity both *in vitro* and *in vivo*.^14^

Herein we exploited the reduction of the oligomers by significantly increasing the rate of Aβ aggregation through specifically targeting its aromatic residues, thus dramatically reducing the toxicity of the peptide. This concept has only been investigated with a few small molecules and a large dendrimer system able to increase Aβ aggregation by non-specific π-stacking interactions,^15–18^ but the use of a macrocycle explicitly binding at the aromatic residues has never been exploited before. Therefore, we investigated the oligomeric nature of Aβ42 in the presence of the eight-membered macrocyclic host molecule cucurbit[8]uril (CB[8], Fig. 1c) and evaluated its toxicity compared to wild type (WT) aggregates. Cucurbituril macrocyclic hosts form stable inclusion complexes in aqueous media and are non-cytotoxic;^19,20^ interactions between CB[8] and aromatic amino acid residues have been widely investigated,^21–27^ especially Phe and Trp, with several recent biochemical applications.^28–30^ Recently, Kim *et al.* ^29^ reported the decrease in aggregation rate of both Aβ40 and Aβ42 in the presence of high concentrations of CB[7], probably on account of the interaction between the macrocycle and the Phe residues in the sequence. Conversely, we focused on CB[8], which is unique in that it is capable of binding two aromatic residues simultaneously in its larger cavity.^23,24^

Aggregates of the Aβ42·CB[8] complexes exhibited similar features to those of the WT, however, they formed much faster and grew much larger in size. CB[8] thus catalyses fast aggregation of the peptide strands surpassing the soluble oligomeric stage, and leads to significantly lower toxicity compared to WT aggregates.

To the best of our knowledge, this is the first report whereby a macrocycle able to specifically target the aromatic residues was employed to induce, rather than inhibit, Aβ42 aggregation in order to decrease its toxicity; we expect our results will give rise to alternative approaches to counter Alzheimer's disease.

## Experimental section

### Peptide synthesis and characterisation

Aβ42 and Aβ42A_4,19,20_ were prepared on a Liberty automated microwave solid-phase peptide synthesiser (CEM) using a Fmoc-based synthetic strategy.^31^ The *ε*-aminohexanoic acid (Ahx, Novabiochem) and fluoresceinisothiocyanate (FITC, Sigma-Aldrich) were then added on a portion of both Aβ42 resins manually; a 5 eq. solution of Ahx/diisopropylcarbodiimide (DIC)/hydroxybenzotriazole (HOBt) was added using a double-coupling strategy and 12 h reaction time, followed by washing and deprotection of the resin with 20% piperidine in dimethylformamide (DMF) (3 times, 3 min each), then a 5 eq. solution of FITC/diisopropylethylamine (DIPEA) 1 : 2 was added and double-coupled for 12 h. To avoid Met oxidation, the cleavage cocktail used to detach the peptides from the resin was reagent H (trifluoroacetic acid (TFA)/thioanisole/phenol/water/ethandithiol/dimethylsulfide/NH_4_I).^32^ After ether precipitation the peptides were stored at –20 °C. The purity of all peptides was assessed by analytical High Pressure Liquid Chromatography (HPLC) (Varian 940-LC) and Electron Spray Ionisation-Mass Spectrometry (ESI-MS, LTQ-Velos), as reported in Fig. S1 and S2 (ESI†). Analytical HPLC was performed using a gradient of acetonitrile from 25% to 90% in mQ water with 0.1%TFA over 17 min (2 min pre-run and 15 min actual run). The column used was a VariTide RPC (Agilent) column 4.6 × 250 mm, with 200 A pore size and 6 μm particles. The peptides were dissolved in 1,1,1,3,3,3-hexafluoro-2-propanol (HFIP, Sigma-Aldrich) and sonicated for 2 min; HFIP was then removed by nitrogen stream and the resulting dry film stored at –20 °C before use.^33^ The monomerisation of the peptides was ensured by dissolution in 60 mM NaOH (1 mg mL^–1^) and then sonicated for 15 min in an ice cold water bath. The resulting solution was passed through a 0.22 μm filter (Millipore) and then through a 10 KDa cut-off Amicon centrifugal filter (Merck Millipore), the concentration was assessed by UV-Vis.^34^ Monomerised peptides were kept on ice and used within 3 h. Unless otherwise stated, the samples were then diluted to the desired concentration using 0.2 μm (Millipore) freshly-filtered 10 mM phosphate buffer (PB), pH 7.4 in the presence or absence of CB[8] at different concentrations and directly measured at time 0 h. Each sample was then incubated at 37 °C with constant rotation (200 rpm) in a shaker incubator (INFORS HT Multitiron II), data were collected every hour until the signal started to decrease on account of peptide precipitation. For transmission electron and atomic force microscopy experiments the samples were incubated at 37 °C with constant rotation (250 rpm) on a Grant Microtube thermo shaker supplied with PSC15 block (Thermo-Fisher).

### Isothermal titration calorimetry (ITC)

To evaluate the binding between the peptides and CBs an ITC-200 (Microcal) was used. A 0.2 μm (Millipore) filtered CB[8] (80 μM) or CB[7] (230 μM) solution was titrated into a freshly prepared peptide (5 μM) solution in 10 mM PB, pH 7.4. The data were collected at 25 °C. After subtracting the blank injection (CB[8] or CB[7] into buffer) values, the data obtained were analysed using Origin software. Each ITC experiment was repeated with three independently prepared samples.

### Thioflavin T (ThT) assay

The ThT assay was carried out to follow the aggregation of Aβ42 in the presence and in the absence of CB[8]; ThT emission was recorded by fluorimeter (Cary Eclipse). The excitation was applied at 450 nm and the emission collected at 495 nm, the temperature was kept at 25 °C. For each data point, a 50 μL aliquot of each sample was added to 750 μL of 0.2 μm (Millipore) freshly-filtered ThT solution (90 μM in PB 10 mM, pH 7.4, Sigma-Aldrich) and the emission collected. The blank emission of ThT in the stated conditions at 495 nm was subtracted and the values normalised to the highest value obtained within the set of experiments. The data are reported as mean ± SD of three independent samples. The fitting is reported in the ESI† (see Tables).^35^

### Circular dichroism (CD)

The transition from a disordered to a β-sheet conformation of Aβ42 in the presence and in the absence of CB[8] was followed by far-UV CD on a Chirascan spectrometer (Applied Photophysics). The data were collected from 250 to 195 nm, using 0.5 nm steps, 1 nm bandwidth, an acquisition time of 1 s and collecting 3 repeats for each sample to minimise the noise, the temperature was kept at 25 °C. Each solution was analysed as three independent samples and smoothed with a factor of 8, the background subtracted and the data averaged.

### Transmission electronic microscopy (TEM)

The fibrils obtained from Aβ42 in the presence and in the absence of CB[8] were recorded by TEM imaging (FEI Philips Tecnai 20). Samples were prepared depositing a 8 μL drop solution at different aggregation times on C300Cu (carbon film on 300 mesh copper) grids (EMResolutions), followed by one water wash and staining using 2% w/v phosphotungstic acid (PTA) aqueous solution.

### Atomic force microscopy (AFM)

The fibrils obtained from Aβ42 in presence and absence of CB[8] were recorded by AFM imaging (Agilent 5500 AFM) using tapping in air mode to further evaluate their morphology. Samples were prepared depositing a 10 μL drop solution after 6 days of incubation on a mica surface (Agar Scientific) approximately 0.5 cm × 1 cm. The surfaces had been previously cleaved in order to remove any extraneous organic matter. The solutions were allowed to remain on the freshly cleaved mica surfaces for 5 min before washing with mQ water (0.6 mL) and allowed to dry in air for at least 30 min. OTESPA-R3 cantilevers (Bruker AFM probes) with a resonant frequency of approx. 314 kHz, tip radius of 7 nm and spring constant 26 N m^–1^ were used. Images were taken over an area of approximately 2 μm^2^ at a rate of 1.02 lines per s with a resolution of 512 × 512 pixels. During measurements the topographic trace in both trace and retrace mode, amplitude and phase were all recorded.

### Dynamic light scattering (DLS)

The aggregation profile of Aβ42 in the presence and in the absence of CB[8] was followed by DLS (Malvern Zetasizer Nano ZS) and the scattered laser light intensities measured at 173° angles, the temperature was set at 25 °C. The data are reported as the mean of three independent samples.

### Cell viability assay

A neuroblastoma cell line, SH-SY5Y (maximum passage used 46), was used to evaluate the toxicity of Aβ42 both in the presence and in the absence of CB[8] *in vitro*. SH-SY5Y cells were cultured with Dulbecco's modified Eagle medium (DMEM) supplemented with 10% fetal bovine serum (FBS) and 1% Pen-Strep (Life Technologies) and incubated at 37 °C and 5% CO_2_. The cells were plated at a concentration 1 × 10^4^ per well on collagen I coated 96-well plates (Life Technologies) and incubated overnight. The toxicity of Aβ42 in the absence of CB[8] was first assessed by treating the cells with media supplemented with different concentrations of Aβ42 freshly dissolved in NaOH (60 mM), as reported in the ESI† (Fig. S8). For experiments in the presence of CB[8], the cell cultures were treated with media containing a fixed concentration of Aβ42 (7 μM), freshly dissolved or incubated as solution in 60 mM NaOH for 24 h at 37 °C with constant rotation (200 rpm) in a shaker incubator (INFORS HT Multitiron II), and different concentrations of CB[8].^36^ In both cases, the cells were tested for mitochondrial activity using a standard MTS, 3-(4,5-dimethylthiazol-2-yl)-5-(3-carboxymethoxyphenyl)-2-(4-sulfophenyl)-2*H*-tetrazolium, assay kit (CellTiter 96 kit, Promega) after 48 h incubation time. A solution of 100 μL phosphate buffered saline (PBS) and 20 μL MTS solution was added to each well and the cells were incubated for 4 h. Cell viability was quantified by measuring the absorbance at 490 nm using a FLASHScan plate reader (Analytik-Jena). The data were averaged from at least three independent replicates and the results were analysed using two-tailed *t*-test to establish any significance between Aβ42 alone and in the presence of different concentrations of CB[8] (see ESI† Tables S2–S4) and the errors are reported as standard deviation at the 95% confidence interval.

### Fluorescence microscopy

The distribution of FITC-Aβ42 *in vitro* was imaged by fluorescence microscopy (Leica TCS SP5) equipped with a digital camera (Leica DFC365 FX). Filter cube A and I3 were respectively used for imaging DAPI and FITC emission. Glass-bottom 48-well plates (SLS) were coated with 10 μg cm^–2^ of rat-tail collagen I (Sigma-Aldrich) and SH-SY5Y cells, cultured as described in the previous section, plated as 3 × 10^4^ cells per well and incubated over night at 37 °C and 5% CO_2_. The cultures were then treated with FITC-Aβ42 freshly dissolved or incubated as solution in 60 mM NaOH for 24 h at 37 °C with constant rotation (200 rpm) in a shaker incubator (INFORS HT Multitiron II) in the presence or in the absence of CB[8] in the culture media. After a 48 h incubation time the cells were fixed with 4% formaldehyde solution in PBS enriched with 4,6-diamidino-2-phenylindole dihydrochloride (DAPI 2 μM, Sigma-Aldrich) to enable DNA staining.

## Results and discussion

### CB[8] induced folding of Aβ42 through aromatic residues complexation

Our previous studies on the dimerisation of Aβ fragments,^24^ encouraged us that CB[8] would exhibit host–guest interactions with the full-length Aβ42. We investigated any interaction using ITC, which demonstrated the occurrence of binding events, as shown in Fig. S3a (ESI†). Fitting sigmoidal curves obtained in the ITC measurements to binding models, however, was elusive suggesting high complexity of the system; nevertheless, valuable information was extracted.

The endothermic peaks for all of the injections (see Fig. S3a, ESI†), suggested that the binding event is entropically driven correlating to a typical folding and aggregation process observed for proteins with a highly hydrophobic core.^37–39^ Moreover, the ITC data resulted in a binding ratio slightly ≥2 for CB[8]:Aβ42, highlighting the presence of several interactions between CB[8] and the three Phe residues (4, 19 and 20), and possibly Tyr_10_. We also carried out ITC of CB[8] with a modified Aβ42 sequence, which contained Ala instead of Phe residues (Aβ42A_4,19,20_) to support the hypothesis of Phe·CB[8] complexation. This ITC data indicated a binding ratio just above 1, likely on account of an interaction with Tyr_10_.^25^ Complexation between CB[8] and Aβ42A_4,19,20_ was noticeably enthalpically driven, suggesting that no aggregation was induced (see Fig. S3b, ESI†). Additionally, ITC of CB[7], which can only bind a single aromatic residue, with Aβ42 was carried out. In this case, exothermic peaks were observed, indicating that indeed no folding was induced during the titration, yielding a curve with a binding ratio close to 4, which corresponds to 1 : 1 binding between CB[7] and the three Phe residues in positions 4, 19 and 20 as well as Tyr_10_ (see Fig. S3c, ESI†). We therefore inferred that the molar ratio observed for the CB[8] titration into the Aβ42 solution is linked to the formation of ternary complexes with Phe residues and a binary complex with Tyr. Importantly, each of the homoternary complexes only account for 0.5 molar equivalents of the complexation observed between CB[8] and Aβ42, whereas the binary complex between Tyr_10_ and CB[8] accounts for 1 equivalent of CB[8]. Considering all of the experimental evidence, CB[8] is likely forming homoternary complexes with the 3 Phe residues, bringing neighboring Aβ42 strands into closer proximity and promoting folding during the ITC experiment.

### CB[8] accelerates Aβ42 aggregation and promotes Phe π–π stacking

**Fig. 2:**
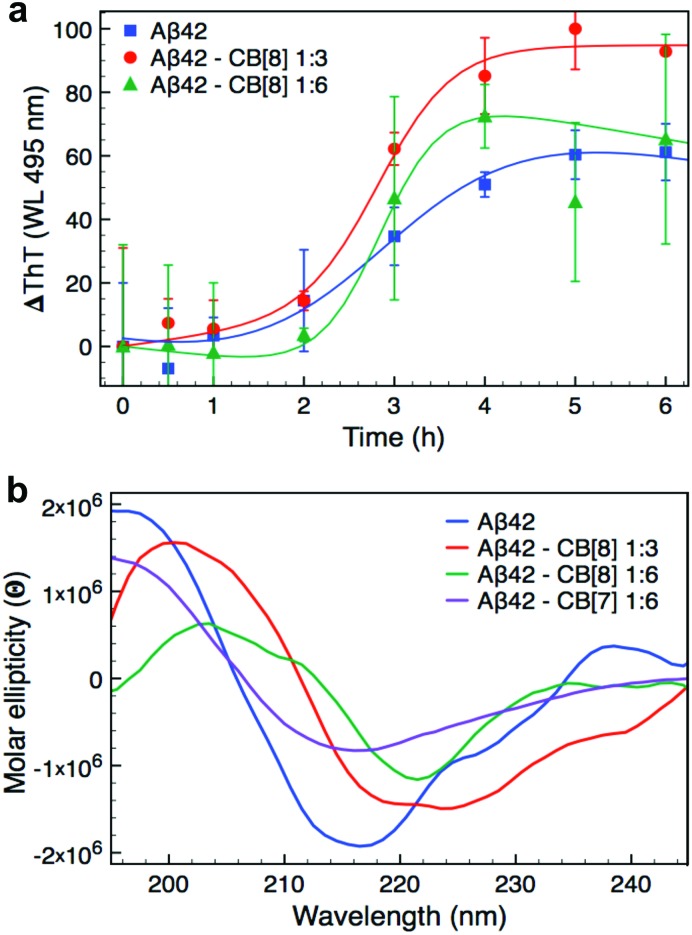
Aggregation studies on Aβ42 and CB[8] complexes. (a) ThT assay of Aβ42 (7 μM) with 0, 3 or 6-fold excess CB[8]. The assay was performed *ex situ* using a final concentration of 90 μM ThT; the data reported are the mean ± SD of three independent samples. (b) CD spectra of Aβ42 (7 μM) with 0, 3 or 6-fold excess CB[8] or 6-fold excess CB[7] after 5 h of incubation. The CD spectrum for Aβ42·CB[8] 1 : 6 was reported after 3 h of incubation, since precipitation was observed over longer periods. All the CD spectra are represented as average of three independent samples. All samples used in the ThT and CD assays were dissolved in 10 mM PB pH 7.4 and directly measured for time 0 h. The samples were then kept at 37 °C and shaken at 200 rpm between each measurement.

After establishing clear binding events between CB[8] and Aβ42, we investigated the influence of CB[8] addition on its aggregation. Firstly, we studied the rate of aggregation using the ThT assay, presented in Fig. 2a. Larger CB homologues can bind ThT as it is an aromatic and positively charged molecule,^40,41^ therefore, we performed the assay *ex situ* in order to avoid any undesired binding of CB[8] to the dye and minimise noise related to this side interaction. The aggregation curves obtained for Aβ42 at different concentrations of CB[8] showed a slightly longer lag phase in the presence of the macrocycle, but a significantly higher rate of elongation, nearly doubling when a 6-fold excess of CB[8] was employed (see Table S1, ESI†). Moreover, the data obtained with a 6-fold excess of CB[8] were extremely noisy, this was probably related to the very high rate of aggregation.

We further investigated the aggregation of Aβ42 both in the presence and absence of CB[8] using far-UV CD to evaluate the change in secondary structure of the peptide (Fig. 2b and Fig. S4 in ESI†). All of the samples exhibited a predominantly disordered conformation upon dissolution with a broad minimum at about 195 nm (time 0 h, Fig. S4, ESI†). After only 1 h, Aβ42 incubated with CB[8] clearly showed bands typical of β-sheet structure, which were absent for Aβ42 incubated alone. The samples with a 6-fold excess of CB[8] reached complete folding within 3 h, whilst Aβ42 samples in the absence of CB[8] required 5 h (see Fig. S4, ESI†). In the presence of CB[8], both the minimum and maximum of the β-sheet bands were shifted from 218 and 196 nm to 220 and 202 nm, respectively (Fig. 2b). This phenomenon has been previously reported, suggesting n–π* interactions between side chains in peptides containing several Phe residues in close proximity.^42–44^ The presence of CB[8] could induce a denser packing of the side chains by simultaneously complexing two Phe residues on neighboring strands.^45,46^ To further explore this structural hypothesis, a far-UV CD experiment was repeated with a 6-fold excess of CB[7]; no red shift was observed (Fig. 2b), corroborating our hypothesis.

### CB[8] increases the size of Aβ42 aggregates and their rate of formation

**Fig. 3:**
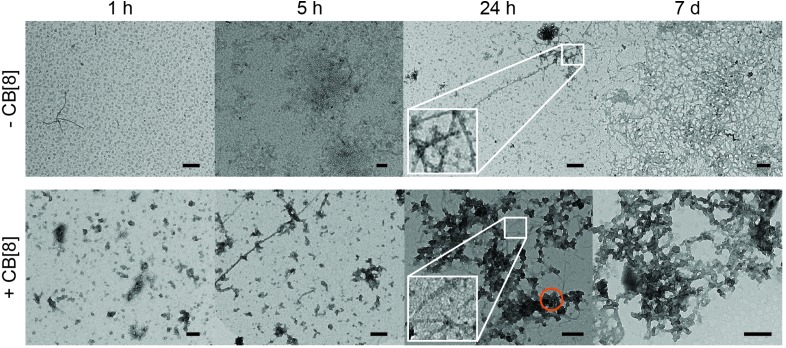
Morphology and size of Aβ42·CB[8] aggregates over time. Aβ42 (7 μM, top) and Aβ42·CB[8] (7 μM and 50 μM, respectively, bottom) samples prepared at different times, from left to right: 1 h, 5 h, 24 h, 7 days. The white boxes at 24 h represent a 9-fold enlargement of the main image both for Aβ42 and Aβ42·CB[8]; the orange circle highlights the presence of globular features in Aβ42·CB[8] sample likely stemming from the interactions between the PTA stain and CB[8]. Each sample was dissolved in 10 mM PB pH 7.4, then incubated at 37 °C and shaken at 250 rpm. All scale bars represent 200 nm.

On account of the increase in aggregation rate of Aβ42 in the presence of CB[8] (ThT assay and time dependent CD measurements), the morphology of the Aβ42·CB[8] aggregates was investigated. TEM was employed to observe the morphology of the negatively stained aggregates at different time points, as depicted in Fig. 3. Although the fibres formed by Aβ42 and CB[8] exhibited a more globular shape (Fig. 3, bottom) compared to those obtained from WT Aβ42 (Fig. 3, top), both samples consisted mainly of oligomers after 1 h of incubation, oligomers and fibres after 5 and 24 h and only fibres after 7 days. It is important to point out that CB[8] alone interacts with the commonly employed stain phosphotungstic acid (PTA), leading to the formation of round particles on the TEM grid (see Fig. S5a, ESI†) similar to the motifs highlighted in the orange circle in Fig. 3. This result suggested that the rounded particles that appear amongst the Aβ42·CB[8] fibres are likely related to the presence of CB[8] localised at the solvent exposed surface of the fibrils. Moreover, in order for CB[8] to interact with the PTA stain and create this globular effect on the TEM grid, one of the two carbonyl-lined portals of CB[8] must likely remain uncomplexed,^47^ suggesting that the macrocycle might bind both Phe residues from the same portal side. This hypothesis is supported by several other examples that have been reported previously where guests preferentially bind with the macrocycle in a *syn* conformation^48,49^ and may be dictated by preferential arrangement of the Aβ42 strands in this case.

As fibre morphology can be inherited from parent seeds,^50,51^ we also added a 10% solution of Aβ42·CB[8] incubated for 24 h to a fresh Aβ42 solution and imaged it by TEM after 6 days in order to evaluate any change in Aβ42 aggregation. The fibres obtained indicated WT Aβ42 features and some of the crosslinks presented a more globular shape, symptomatic of CB[8] presence (see Fig. S5b, ESI†), thus the Aβ42·CB[8] complexes are likely behaving as on-pathway aggregates.

In an effort to characterise the fibres formed both in the presence and in the absence of CB[8] without interference from any staining, AFM was used. As reported in ESI† Fig. S6, WT Aβ42 and Aβ42·CB[8] samples presented both fibrils (several hundred nm in length and approximately 1 nm in height) and longer fibres (>1 μm in length). The main difference recorded was in the height of the fibres: 2.0 nm maximum for Aβ42·CB[8] fibres, whereas WT Aβ42 fibres showed a height around 4 nm. The observed difference in height supports a similar protofibrillar structure to that of the WT, but with a different assembly of the protofibrils into mature fibres.^5,52,53^

In order to evaluate the increase in size of the aggregates, their hydrodynamic radius was measured by DLS, as reported in Fig. S7 (ESI†). The data were collected over time to closely follow the change in dimension of the complexes. Aβ42 alone showed a polydisperse population, with some small particles (12 nm) and larger aggregates (80 nm) from the first measurement; an increase in size over 6 h up to 700 nm in diameter was observed (see Fig. S7a, ESI†). Samples containing CB[8] were also polydisperse, but the size of the aggregates appeared to be larger than Aβ42 alone; in the presence of a 6-fold excess of CB[8] the major populations upon dissolution (time = 0 h) were already 21.5 and 380 nm (see Fig. S7c, ESI†). The size of the aggregates in the Aβ42·CB[8] samples increased more rapidly than in the samples without CB[8] and the final distribution after 6 h showed high polydispersity with larger aggregates ranging from 600 to 1200 nm (see Fig. S7, ESI†).

The ThT assay indicated a longer lag phase for the Aβ42·CB[8] complex than for WT Aβ42, but an increase in the rate of elongation. The data obtained by CD, however, demonstrated an earlier conversion to β-sheet structure and, in conjunction with TEM and DLS data, showed a faster rate of aggregation for Aβ42·CB[8]. The early aggregates formed in the presence of CB[8] can be considered on-pathway, as in the presence of WT Aβ42, they further assemble to form fibres that retain the major structural characteristics of the WT, while incorporating several features observed in the Aβ42·CB[8] system (Fig. S5b, ESI†). Nevertheless, the structural differences inherent to the soluble oligomers (in the presence of CB[8]) produce fibres, which are generally more cross-linked and have different heights, as reported by TEM and AFM studies, suggesting that Aβ42·CB[8] aggregates pack differently into mature fibres. Considering all the data collected, it is evident that CB[8] enhances the aggregation rate of Aβ42, producing fibrillar aggregates of a larger size compared to those of the WT.

### Aβ-CB[8] aggregates are less toxic and show lower cell-uptake

**Fig. 4:**
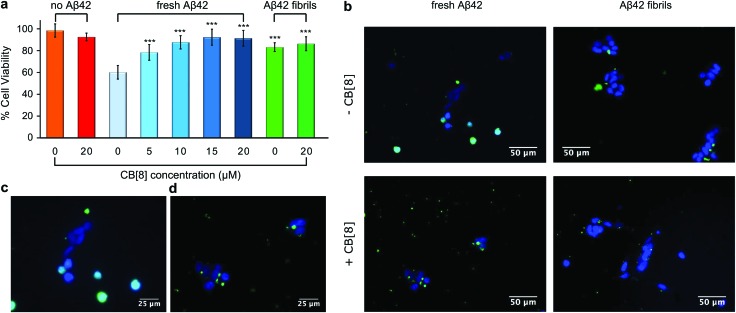
Cell viability data and fluorescence microscopy imaging. (a) Cell viability data collected on a SH-SY5Y cell line using the MTS assay. Media supplemented with Aβ42 (7 μM) in the presence of different concentrations of CB[8] was added to the cell culture. The cells were incubated for 48 h before the viability assay was performed. The data reported are the mean ± SD of at least three independent plates. The significance for each data compared to fresh Aβ42 was at least *p* < 0.001 (***). (b) Fluorescence microscope images of SH-SY5Y cells incubated for 48 h with media supplemented with FITC-Aβ42 (10 μM) in the presence and absence of CB[8] (20 μM). Higher magnification images of SH-SY5Y cells incubated for 48 h in the presence of (c) FITC-Aβ42 fresh or (d) FITC-Aβ42 fresh·CB[8]. FITC-Aβ42 fresh denotes that the peptide was added to the media upon dissolution, while FITC-Aβ42 fibrils denotes that the peptide was incubated at 37 °C and shaken at 200 rpm for 24 h in NaOH (60 mM) before addition to the cell media.

In order to evaluate whether or not the structural differences between the aggregates formed by Aβ42 in the presence and in the absence of CB[8] are associated to different toxicity profiles, freshly dissolved Aβ42 in the presence of varying concentrations of the macrocycle was added to SH-SY5Y culture media. While WT Aβ42 showed an inherent toxicity after 48 h of incubation by MTS assay, the use of 1 equivalent of CB[8] significantly increased the cell viability, which reached a maximum value with only 1.5 equivalents of CB[8] (Fig. 4a). On account of the difference in toxicity, we employed fluorescence microscopy to image oligomer distribution within the cells. In order to have a dye directly attached to the peptide, which did not interact with the macrocycle, we prepared an used an N-terminal FITC-labelled Aβ42 (FITC-Aβ42) in these experiments. As Aβ oligomers interact with cell membranes,^5^ we expected to image fluorescence inside SH-SY5Y cells after incubation for 48 h in cell media containing FITC-Aβ42. Bright field images (see Fig. S9, ESI†) showed that the majority of SH-SY5Y cells had lost their characteristic shape becoming rounded, thus indicating cell death. Extensive FITC fluorescence was observed throughout these dead cells, as shown in Fig. 4b and c, and its overlap with DAPI fluorescence demonstrated the catastrophic disruption of the nuclear envelope. The few live cells that remained after 48 h did not present any significant FITC fluorescence. On the contrary, when FITC-Aβ42 was added to the cells in the presence of CB[8], the FITC emission was distributed both outside the cells and in the cytoplasm of living cells, but not in the nuclei (stained blue with DAPI) (Fig. 4b and d). Moreover, the FITC fluorescence in the cytoplasm did not appear to be homogeneously distributed, suggesting that the uptake of these supramolecular oligomers happens through a different pathway, possibly endocytosis, and their toxicity is thus substantially lower.

To assess the impact of any possible interactions between a pre-aggregated Aβ42 and CB[8] on cell viability, a peptide solution was incubated for 24 h at 37 °C to provide fibrils, which were then added to SH-SY5Y culture media with and without the macrocycle. Although the data obtained by MTS assay showed a higher cell viability than fresh WT Aβ42, the values in the presence and in the absence of CB[8] were indistinguishable (*p* > 0.05, Fig. 4a). Nevertheless, they suggested that either CB[8] is unable to interact with pre-aggregated Aβ42 fibrils, or the species formed by fibrillar Aβ42 and CB[8] do not exhibit a significant toxicity. The cell uptake behavior of the fibrillar FITC-Aβ42 was the same irrespective of the presence of CB[8], but differed from the fresh FITC-Aβ42 uptake; however, these fibrils mirrored the uptake observed for fresh FITC-Aβ42 when incubated with the cells in the presence of CB[8] (Fig. 4b). These results suggested that the faster aggregation induced by CB[8] is indeed directly correlated with a lower *in vitro* toxicity.

## Conclusion

One of the major challenges hindering a detailed understanding of the Aβ toxicity mechanism is to define its toxic structure on account of the peptide's ability to constantly change conformation in a fast equilibrium among different oligomers. Most of the studies in the literature have been focusing on the complete inhibition of the aggregation of Aβ,^1,5^ while only a few have suggested increased aggregation as a possibility for toxicity reduction.^15–17^ Recently, the inhibition of one specific aggregation pathway that impedes oligomer production, yet does not prevent fiber formation, has been reported^14^.

**Fig. 5:**
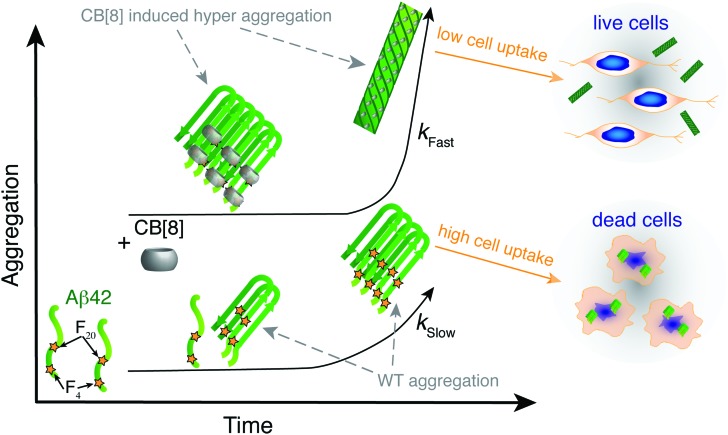
CB[8] modifies exposure of hydrophobic residues and aggregation rate, thus reducing Aβ42 toxicity. Schematic representing the aggregation of Aβ42 over time in the presence and in the absence of CB[8]. When the macrocycle is added to Aβ42, the hydrophobic residues are shielded and the aggregates grow faster and are larger, thus reducing Aβ42 cell uptake and toxicity.

In the present study we employed the macrocycle CB[8] to simultaneously complex aromatic residues from adjacent strands of Aβ42 to modify the aggregation propensity of the sequence (Fig. 5). The aggregates formed are able to surpass the oligomeric toxic stage ensuring higher cell viability. CB[8] clearly demonstrated preferential binding to the Phe residues and, most likely, to Tyr_10_ as well, thus altering the solvent exposure of these residues. The aggregates formed by Aβ42 and CB[8] enhanced ThT fluorescence, confirming the formation of hydrophobic pockets, and they also exhibited β-sheet formation by CD at a very early stage of aggregation (<1 h). Importantly, the CD bands obtained from the supramolecular assemblies showed a red shift compared to WT Aβ42, indicating a denser packing of Phe residues, complexed within the CB[8] cavity.^42–44^ Additionally, ThT, CD, TEM and DLS data suggested that CB[8] was able to significantly increase the rate of Aβ42 aggregation, which is in agreement with our theory (Fig. 5).

Most importantly, Aβ42 showed a lower toxicity in a neuronal cell line in the presence of CB[8]. Moreover, fluorescence imaging showed a lower cell uptake of Aβ42 administered in conjunction with CB[8] than in its absence. These results might be related to several factors, such as a different interaction with the cell membrane in the presence of the macrocycle, but are clearly related to an increased rate of aggregation that enables the formation of larger aggregates in a shorter period of time, which appear to be less prone to cell uptake.

The use of a supramolecular host, the macrocycle CB[8], decreases the *in vitro* toxicity of Aβ42 by increasing its rate of aggregation, in contrast to the inhibition-mediated approach commonly adopted. Our findings pave the way for alternative routes to counteract Alzheimer's disease, exploiting supramolecular chemistry.

## Conflict of interest

The authors declare no competing financial interest.
