# Supplementary material for: Decreasing amyloid toxicity through an increased rate of aggregation
Source: Phys Chem Chem Phys. 2016 Nov 30;19(2):1458–65. doi: 10.1039/c6cp06765d (PMC5310522; doi:10.1039/c6cp06765d)
Supplement: Supplementary file 1 [file CP-019-C6CP06765D-s001.pdf]

Electronic Supplementary Material (ESI) for Physical Chemistry Chemical Physics.
This journal is © the Owner Societies 2016

Supplementary Information for: Decreasing amyloid toxicity through an increased rate of aggregation

Silvia Sonzini, Helen F. Stanyon, and Oren A. Scherman*

Melville Laboratory for Polymer Synthesis, Department of Chemistry, University of Cambridge, Lensfield Road, Cambridge, CB2 1EW, United Kingdom

E-mail: oas23@cam.ac.uk

Chemicals and Reagents

All amino acids used, hydroxybenzotriazole (HOBt), dichloromethane (DCM), dimethyl formamide (DMF) and 20% piperidine in DMF solution were bought from AGTC Bioproducts and used without further purification. NovaPEG Rink amide LL resin and ϵ-aminohexanoic acid (Ahx) were purchased from Merck Novabiochem. Diisopropylcarbodiimide (DIC), diisopropylethyl-amine (DIPEA), trifluoroacetic acid (TFA), diethyl ether, thioanisole, ammonia iodide, thioflavin T (ThT), fluoresceinisothiocyanate (FITC), monobasic potassium, dibasic sodium phosphate and phosphotungstic acid (PTA) were purchased from Sigma–Aldrich. HPLC–grade acetonitrile (ACN) and *N*–methylpyrrolidone (NMP), phenol, dimethylsulfide (DMS), ethandithiol (EDT) and analytical grade purity 1,1,1,3,3,3,-hexafluoroisopropanol (HFIP) were bought from Fisher Scientific. The buffer used for the photophysical characterizations was a sterile filtered 10 mM phosphate buffer (PB) adjusted to pH 7.4 prepared by dilution from a stock solution of 0.5 M phosphate buffer pH 7.4 in ultrapure water

(Synergy UV Ultrapure water system). CB[8] and CB[7] were synthesised following a published procedure[1] and their concentrations were standardized by calorimetric titration with methyl viologen (Sigma Aldrich). CB[8] concentration in the cell media was verified by UV-titration.[2]

Thioflavin T Assay

The data from the ThT assay were fitted using an empirical equation previously reported:[3]

$$Y = (y_i + m_i x) + \frac{(y_f + m_f x)}{1 + e^{-(x-x_0)/\tau}} \tag{S1}$$

where Y is the fluorescence intensity, x is the time, x_0 is the time at half-height of fluorescence (t_{50}), and τ is a time constant. From this equation some empirical parameters can be obtained, such as the lag time (t_{lag} = x_0 - 2τ), the apparent fibre growth rate (k_{app} = $1/\tau$), and the t_{50}. The values we obtained are reported in S1.

Table S1: Values obtained for t_{lag} and k_{app} from fitting the ThT assay data to equation S1 for Aβ42 with 0, 3 or 6-fold excess CB[8].

CB[8] equivalents	0	3	6
t_{lag} (h)	1.26	2.02	2.16
k_{app} (h^{-1})	1.26	2.41	2.94

Cell Viability Assay

In order to establish the significance of the data obtained for the MTS assays on cells treated with Aβ42 and different concentrations of CB[8], we analyzed the results with the two-tailed paired t-test; the P-values obtained are reported in the following tables (S2 and S3).

Table S2: p-values from MTS assays on SH-SY5Y cells treated with media containing different concentrations of CB[8] and supplemented with freshly dissolved Aβ42.

p-value	CB[8] 5 μM	CB[8] 10 μM	CB[8] 15 μM	CB[8] 20 μM
. CB[8] 0 μM	2.99×10^{-4}	1.26×10^{-7}	4.06×10^{-8}	6.47×10^{-8}
CB[8] 5 μM	—	0.046	0.072	0.011
CB[8] 10 μM	—	—	0.306	0.411
CB[8] 15 μM	—	—	—	0.844

Table S3: p-values from MTS assays on SH-SY5Y cells treated with media supplemented with Aβ42 fibrils in the absence and in the presence of CB[8] (20 μM) compared with freshly dissolved Aβ42.

p-value	Aβ42 fibrils - CB[8] 0 μM	Aβ42 fibrils - CB[8] 20 μM
Aβ42 fresh CB[8] 0 μM	1.58×10^{-6}	1.20×10^{-6}

Additional Figures

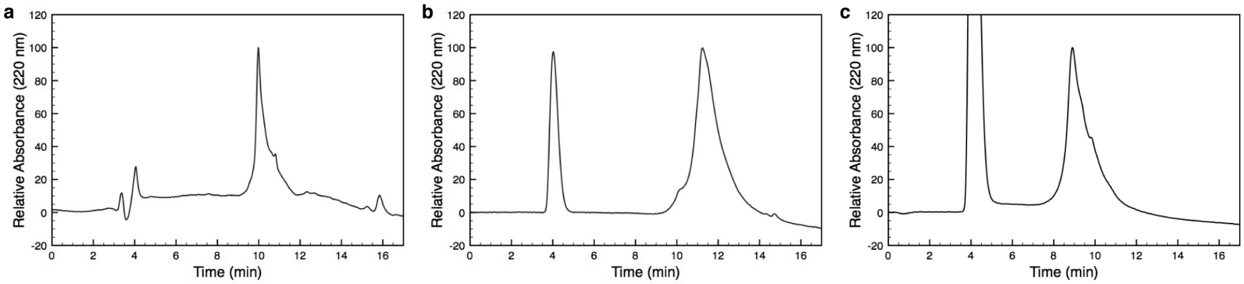

Figure S1: HPLC traces. a) HPLC trace of Aβ42, b) FITC-Aβ42 and c) Aβ42A$_{4,19,20}$. All the traces are reported for the absorbance at 220 nm. In each figure the peak at 4 min represents the injection peak.

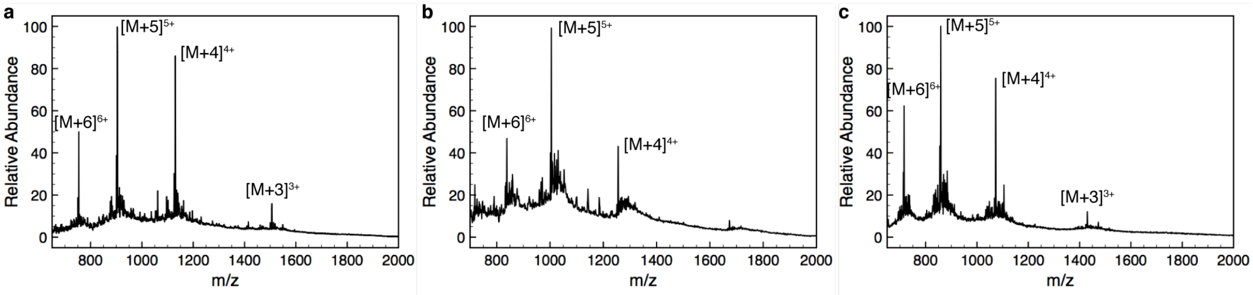

Figure S2: ESI analyses. a Aβ42, MW: 753.84 (M+H)$^{+6}$, 904.34 (M+H)$^{+5}$, 1130.09 (M+H)$^{+4}$, 1506.10 (M+H)$^{+3}$ (theor. MW: 4513); b) FITC-Aβ42, MW: 837.59 (M+H)$^{+6}$, 1004.92 (M+H)$^{+5}$, 1255.76 (M+H)$^{+4}$ (theor. MW: 5020); and c) Aβ42A$_{4,19,20}$, MW: 715.76 (M+H)$^{+6}$, 858.68 (M+H)$^{+5}$, 1073.01 (M+H)$^{+4}$, 1430.02 (M+H)$^{+3}$ (theor. MW: 4285).

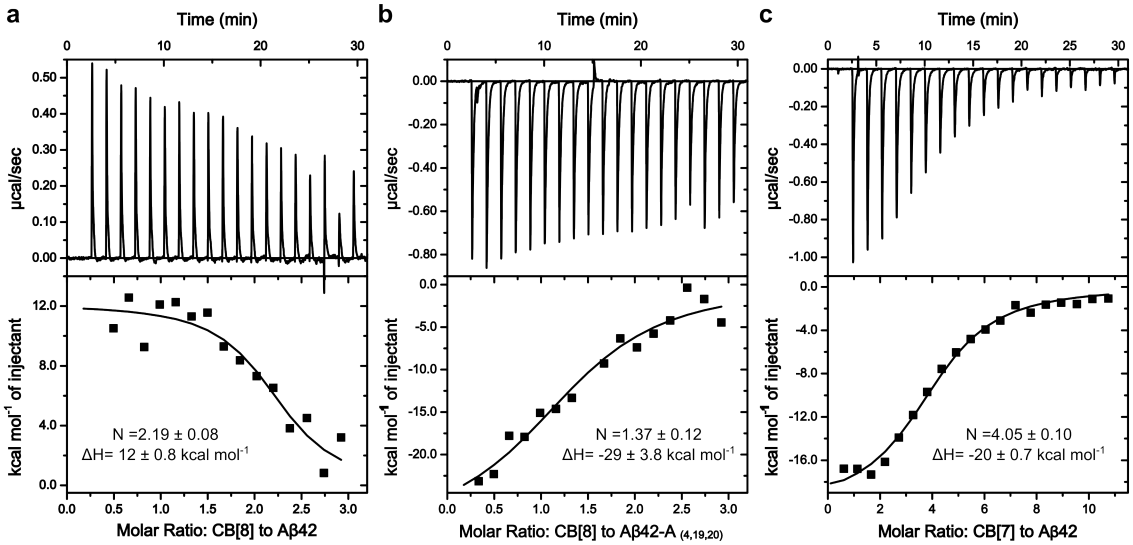

Figure S3: Binding analyses. a) ITC of CB[8] (80 μM) titrated into Aβ42 solution (5 μM) and b) Aβ42A$_{4,19,20}$ (5 μM). c) ITC of CB[7] (280 μM) titrated into Aβ42 solution (5 μM). All the samples were dissolved in 10 mM PB pH 7.4 and immediately measured. Each experiment has been repeated at least three times.

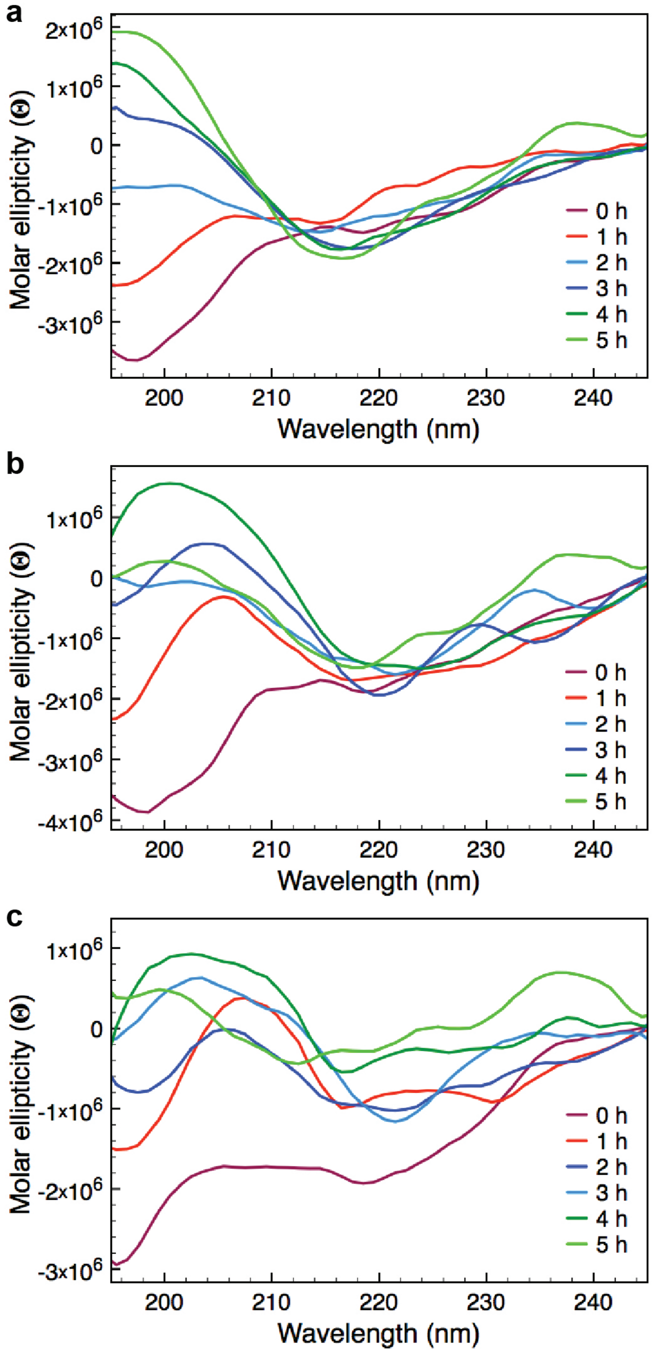

Figure S4: CD analyses over time. a-c) CD spectra of Aβ42 (7 μM) with 0, 3 or 6-fold excess CB[8] respectively showing aggregation over time. All the CD spectra are represented as average of three independent samples. All the samples were dissolved in 10 mM PB pH 7.4 and directly measured for time 0 h. The samples were then kept at 37 °C and shaken at 200 rpm between each measurement

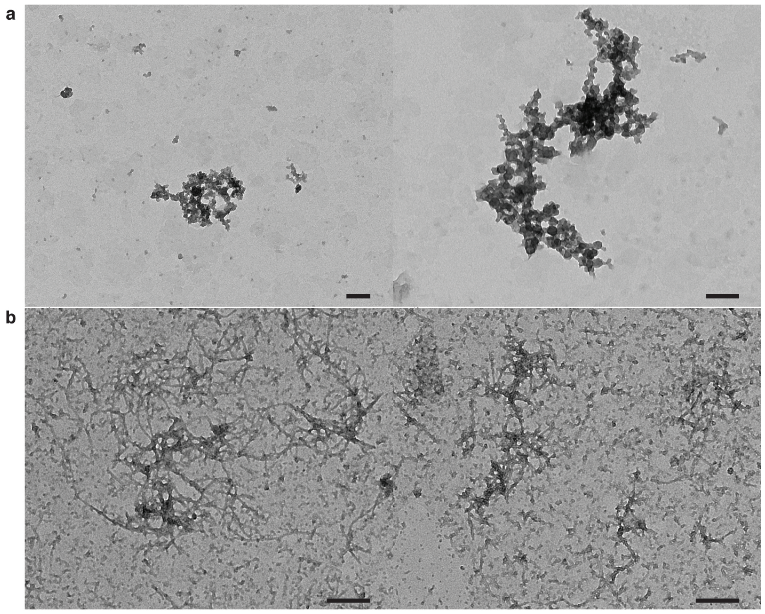

Figure S5: Additional TEM images. a) TEM images of CB[8] only. b) TEM images of Aβ42 (80 μL of 7 μM solution) seeded with a 10 μL solution of Aβ42·CB[8] (7 μM and 50 μM, respectively) pre-incubated for 24 h at 37 °C and shaken at 250 rpm. The samples were then incubated for an additional 6 days before imaging. The samples were all dissolved in 10 mM PB pH 7.4. Scale bars represent 100 nm.

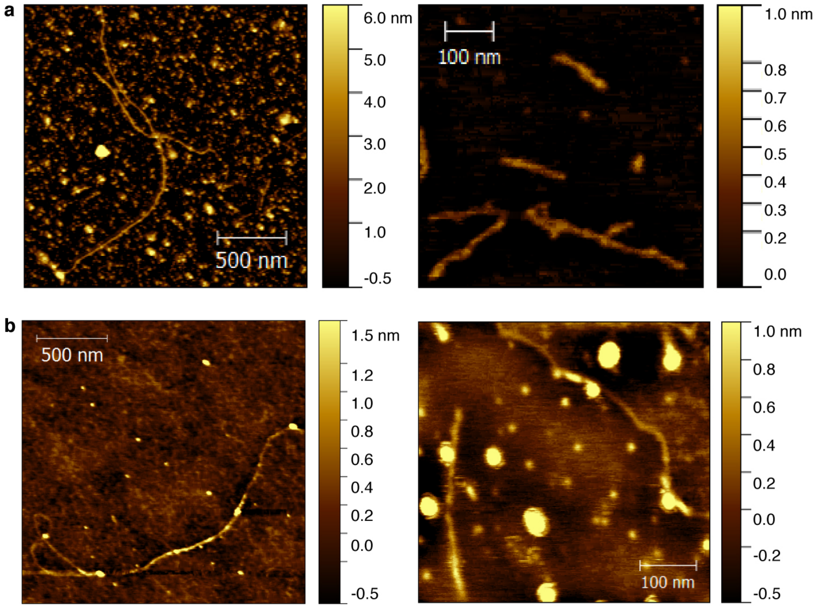

Figure S6: AFM topography images of Aβ42 with and without CB[8]. a) Aβ42 (10 μM) and b) Aβ42·CB[8] (10 μM and 50 μM, respectively) samples. All samples were dissolved in 10 mM PB pH 7.4, then incubated at 37 °C and shaken at 250 rpm for 6 days.

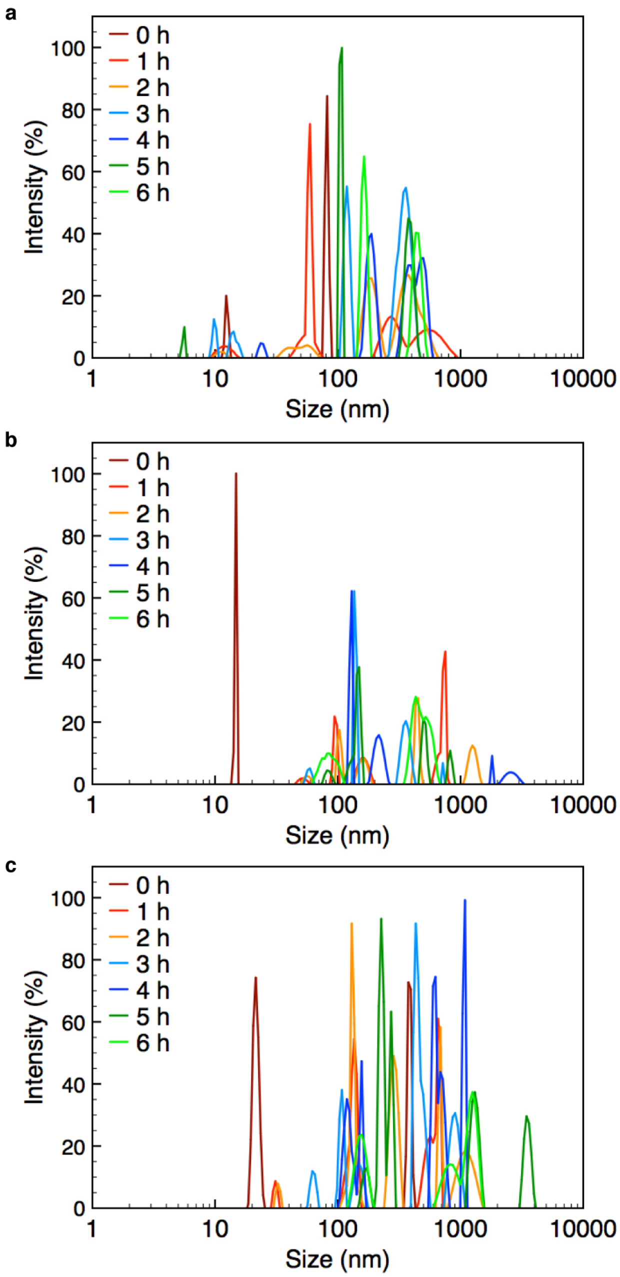

Figure S7: DLS measurements over time. DLS intensity data of a) Aβ42 (7 μM), b) Aβ42·CB[8]1 :3 (7 μM and 22 μM, respectively) and c) Aβ42·CB[8]1:6 (7 μM and 45 μM, respectively). DLS samples were dissolved in 10 mM PB pH 7.4 and directly measured for time 0 h. The samples were then kept at 37 °C and shaken at 200 rpm between each measurement. The data were averaged from at least three independent samples.

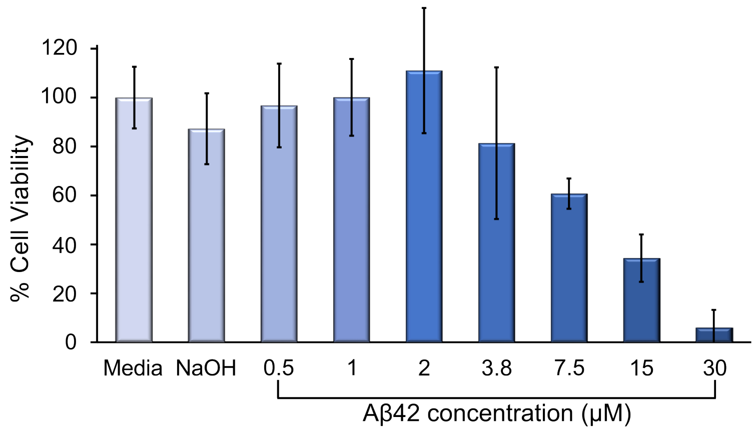

Figure S8: Cell viability data collected on the SH-SY5Y cell line using the MTS assay. Freshly dissolved Aβ42 was added at different concentrations to the cell media. The cells were incubated for 48 h before the viability assay was performed. The data reported are the mean $\pm$SD of at least three independent plates.

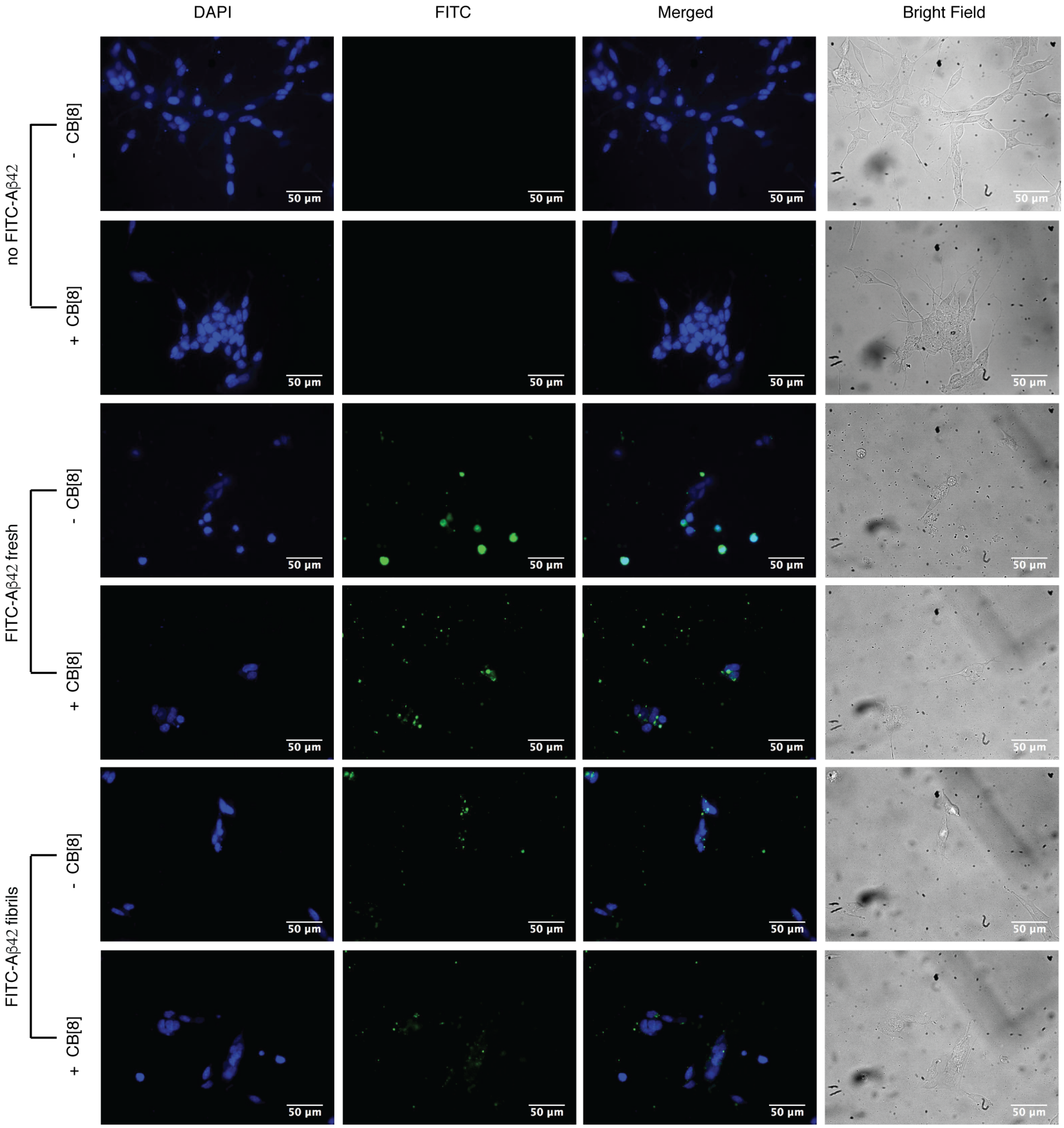

Figure S9: Fluorescence microscope images of SH-SY5Y cells incubated for 48 h in different conditions. From top to bottom: media only, CB[8] only (20 μM), FITC-Aβ42 fresh (10 μM) with and without CB[8] (20 μM), FITC-Aβ42 pre-aggregated for 24 h at 37 °C and shaken at 250 rpm (10 μM) with and without CB[8] (20 μM).

References

(1) Lee, J. W., Samal, S. Selvapalam, N., Kim, H.-J., Kim, K. (2003) Cucurbituril homologues and derivatives: New opportunities in supramolecular chemistry. *Acc. Chem. Res. 36*, 621–630.

(2) Yi, S., Kaifer, A. E. (2011) Determination of the Purity of Cucurbit[n]uril (n = 7, 8) Host Samples *J. Org. Chem. 76*, 10275–10278.

(3) Nielsen, L. *et al.* (2011) Effect of Environmental Factors on the Kinetics of Insulin Fibril Formation: Elucidation of the Molecular Mechanism. *Biochemistry 40*, 6036–6046.
